# Direct current galvanic vestibular stimulation modulates sound localization abilities

**DOI:** 10.1038/s41598-025-92064-y

**Published:** 2025-03-01

**Authors:** Assan Mary Cedras, Clara Orsini, Daniel Paromov, Benoit Antoine Bacon, François Champoux, Maxime Maheu

**Affiliations:** 1 Faculty of Medicine, School of Speech-Language and Audiology, University of Montreal, C.P. 6128, Succursale Centre-Ville, 7077 Avenue du Parc, bureau 3001-42, Montreal, QC H3C 3J7 Canada; 2 Department of Psychology, The University of British Columbia, Vancouver, BC Canada; 3 Centre de recherche de l’institut universitaire de gériatrie de Montréal (CRIUGM), Montreal, QC Canada; 4 Institut universitaire sur la réadaptation en déficience physique de Montréal (IURDPM), Pavillon Laurier, CIUSSS du Centre-Sud-de-l’île-de-Montréal, Montreal, Canada

**Keywords:** Audio-vestibular interaction, Galvanic vestibular stimulation, Sound localization, Spatial auditory encoding, Sensory processing, Perception

## Abstract

The vestibular system has been shown to play a role in the integration of spatial sensory information. For instance, vestibular perturbations induce significant shifts in spatial tactile tasks, but results have been contradictory regarding auditory modality. This observation may be because some of the previous vestibular stimulation methods (i.e., stochastic GVS) did not reliably induce a self-motion effect. This study aims to evaluate the importance of directional illusory motion on auditory localization mechanism, using direct current GVS. Twenty young healthy participants performed a sound localization task under earphones with 9 positions in the azimuth plane divided into three quadrants 7Left (45°;− 30°;− 20°), Center (− 10°;0°;10°), and Right (20°;30°;45)]. Participants were asked to verbally identify the exact position of the sound source under 3 conditions: (1) Without GVS (2) GVS with anode on the right mastoid (3) GVS with anode on the left mastoid. Results were analyzed using the non-parametric Friedman test and Wilcoxon rank-sum post-hoc test with a Bonferroni correction applied to account for multiple comparisons. Compared to baseline, left anodal stimulation caused a greater error ratio for sounds in all quadrants. Moreover, for sounds in the right quadrant, a significantly greater error ratio was observed for anode left compared to the anode right condition. Right anodal condition caused a greater error ratio for sounds in the left and the center quadrants compared to the baseline condition. This study demonstrates for the first time, that sound source localization can be influenced by direct current GVS and is modulated according to the anode position.

## Introduction

The ability to adequately represent our body in space is crucial for several daily activities such as navigating our environment. Many sensory systems, such as the somatosensory system, the visual system, the vestibular system, and the auditory system, have been demonstrated to participate in the representation of the body in space^1,2^. Interestingly, it has been proposed that vestibular signals are necessary for other sensory cues to be properly integrated and to provide an accurate and reliable extra personal space representation^3^. Indeed, there is behavioral evidence that a perturbation of the vestibular afferent signals modulates both visual and somatosensory spatial representation. More specifically, it has been demonstrated that vestibular stimulation (vestibular caloric stimulation; galvanic vestibular stimulation), depending on the signal, could perturb spatial visual encoding in healthy participants^4^, or improve visual hemineglect in right hemisphere lesioned patients^5,6^. A similar asymmetric influence was also observed for a somatosensory task in hemianesthesia^7^, where it was observed that a left cold vestibular caloric stimulation improved both left and right hemianesthesia. The observed effects, both on visual and somatosensory spatial encoding, seem to be dependent on the ear being stimulated as left visual hemineglect and hemianesthesia patients’ performance was improved by either increasing vestibular afferents stimulus rate to the right ear (right warm caloric; cathode right) or decreasing vestibular stimulus rate to the left ear (left cold caloric; anode left)^5–7^.

A recent study suggests that a change in the orientation of the body in space, without awareness that body orientation was altered, could lead to a significant illusory shift in the localization of a sound source^8^, underlining the significance of multisensory integration for body representation and its impact on auditory localization. It has also been demonstrated that vestibular stimulation can perturb spatial auditory encoding in healthy participants following rotational or caloric stimulations^9–11^. Indeed, early experiments demonstrated that a change in auditory localization relative to the body can be observed following rapid rotational deceleration, which caused subjects to localize sound with a bias towards the direction of the preceding rotation^9^.

Additionally, the modulatory effect of cold caloric or whole-body rotatory acceleration on sound localization has also been observed^10,11^. These authors found a bias towards the contralateral side of the ear being stimulated with cold caloric vestibular stimulation^10^, and towards the direction of rotation following passive whole-body rotation about the earth-vertical axis^11^. However, a recent study that investigated the use of stochastic galvanic vestibular stimulation (GVS) to explore the impact of vestibular perturbation on auditory localization did not reveal an impact of stochastic GVS on the ability to encode the spatial position of the sound source^4^. Therefore, to date, a direct influence of vestibular perturbation on auditory spatial encoding remains putative or debated. One hypothesis to explain the absence of influence of GVS in Zanchi et al.^4^ could be the absence of clear directional illusory self-motion perception of the signal used and the subsequent failure to update the representation of the body midline in space. In fact, these authors reported that their GVS signal induced “a sense of instability without consistent or directional illusory motion.

Contrary to the galvanic vestibular stimulation described in Zanchi et al.^4^ direct current vestibular stimulation is well-known to induce a sense of rotation around Reid’s plane^13,14^ which leads to a clear illusionary shift of our position in space. Therefore, the objective of the present study is to determine the importance of directional illusory motion on auditory localization mechanism, using direct current GVS. The results could also help deepen our understanding on the mechanisms responsible for audio-vestibular interactions.

## Methods

### Participants

Twenty healthy participants were recruited, but only 17 were included in the analysis as three could not tolerate GVS [11 females; 15 right-handed; mean age: 23.82 ± 2. 22 years old] (see Table 1. for demographic characteristics). No other participants experienced significant adverse effects except from the three participants that reported being unable to tolerate the GVS sensation. Exclusion criteria included a previous history of ear surgery, dizziness, uncontrolled diabetes, cervical, neurological, or ocular motor pathologies. All subjects provided written informed consent before testing. This study was approved by the ethics committee of the University of Montreal (CERC-2022-1746) and met the requirements of the Declaration of Helsinki. All methods were performed in accordance with the relevant guidelines and regulations.

**Table 1:** Demographic characteristics (sex, age, and handedness) of the participants included in the present study.

Participants (n = 17)	Men	6
	Women	11
Handedness (n = 17)	Right-handed	15
	Left-handed	2
Age	Mean	23.824
	Std deviation	2.215

### Protocol

All participants first underwent hearing and vestibular screening. The hearing screening included an otoscopic examination, tympanometry, and pure-tone audiometry from 0.25 to 8 kHz. A thresholds within normal limits (≥ 20 dBHL) from 0.25 to 8 kHz, bilateraly was used as inclusion criteria. For the vestibular evaluation, the video Head Impulse Test (vHIT; ICS impulse; Otometrics; Denmark) was used, as described by Halmagyi et al.^15^ to test the function of the horizontal canals. A gain superior of 0.8 and an absence of saccades were used as inclusion criterias. Participants then were asked to perform the sound localization task under 3 conditions: (1) Baseline (without GVS), (2) GVS-Anode right (anode on the right mastoid and cathode on left mastoid), (3) GVS-Anode left (Anode on the left mastoid and cathode on right mastoid). In order to verify the participant’s ability to locate sounds with precision (i.e., task comprehension), the experimentation began with a familiarization period and a trial run. Conditions were presented in a randomized order except for the baseline, which was done first. This order was selected to avoid any possible bias on the performance at the baseline condition due to possible prolonged effect of GVS.

### Galvanic vestibular stimulation

Galvanic vestibular stimulation (DC-Stimulator, NeuroConn) was used with two electrodes (anode and cathode) of 3 cm^2^ placed on both mastoids, transmitted a transcranial direct-current stimulation of 2 mA. The GVS began before (to allow for the ramp on) and lasted throughout the auditory localization task (approximately 5 min). The electrodes were placed at the beginning of the experimental trial and were turned on and off, depending on the condition. As head pitch position has been demonstrated to modulate the perceived rotation induced by GVS, participants were lying on their back on a bed elevated at 30⁰ from the horizontal plane in order to reduce the variability of the perceived sensation of rotation in the roll plane between trials and participants^13^. In addition to being in a room where the lights were out, participants were wearing videonystagmography goggles (VNS3X, Synapsis) preventing any visual cues except for a centered red light that allowed for the suppression of nystagmus that may be caused by GVS.

### Auditory localization task

**Fig. 1:**
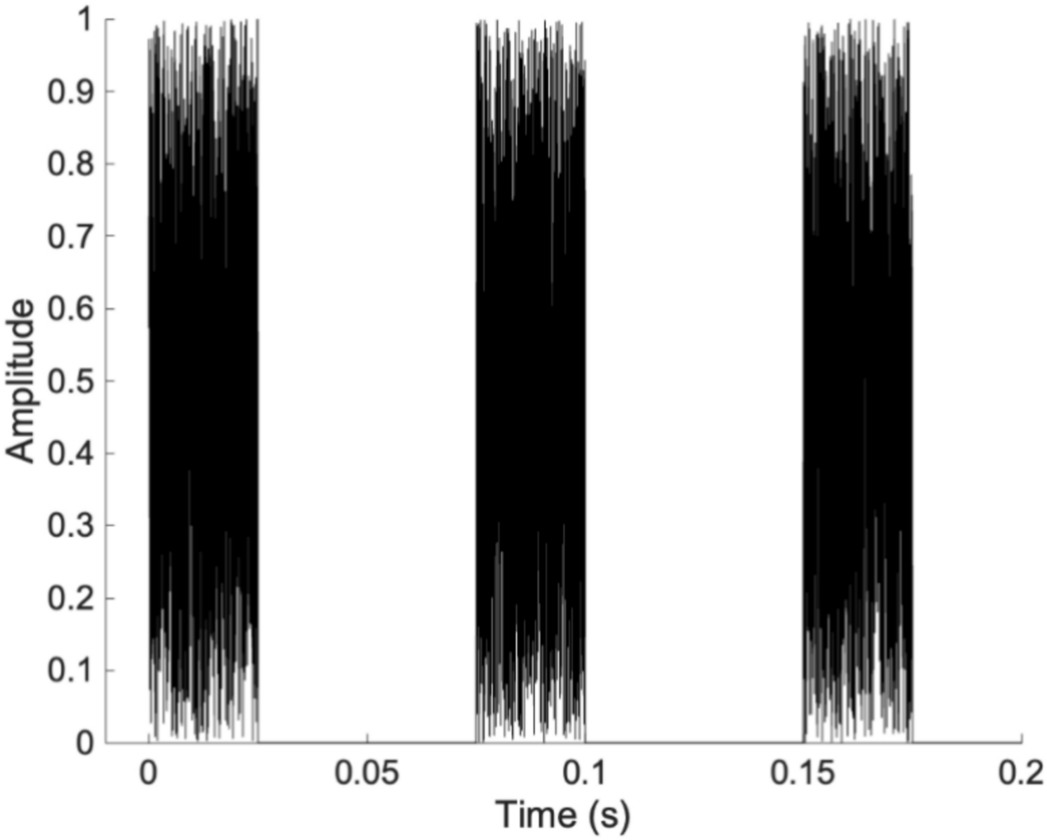
Representation of the click stimuli separated by a 50 ms pauses.

White noise bursts generated by Matlab (R2020a) were presented to the participants through a soundcard (U-Phoria UMC202HD, Behringer, Germany) and inserts-earphones (Eartone, 3A inserter Earphone). The stimulus consisted of 3 bursts each of a 25 ms duration, separated by 50 ms pauses (Fig. 1). The use of inserts-earphones allowed to maintain stable auditory cues even if participants moved their heads in response to GVS signal. Participants had to verbally report the localization of the sound by choosing between eleven (11) simulated sound positions (90°, 45°, 30°, 20°, 10° at the right or left side, as well as 0°) created by the 3-D Head Related Transfer Function of Matlab (R2020a) which is based on the SOFA files defined by the Audio Engineering Society (AES69). The sound intensity for a sound presented at 0° was measured at 53dBA bilaterally using a SoundAdvisor™ Sound Level Meter Model 831C (Larson Davis, Depew, NY, USA).

In order to assess the participant’s ability to appropriately complete the task, a familiarization task and trial run were completed before the experimental conditions. The familiarization task consisted of a presentation of the 11 sound positions through the headphones with the experimenter verbally indicating to the participant the spatial position relative to body midline (in degrees) of the sound source. The experimenter explicitly explained to the participants that no sounds beyond ± 90° would be presented. Afterwards, a trial run was completed where the participant had to verbally report the perceived position of the stimulus, and the experimenter confirmed or corrected the response of the participant. The participants’ performance was not computed. This ensured that the participant was able to correctly identify sounds sources positions without front-to-back or left–right confusion. For the experimental trials (following presentation and practice trials), six blocks of stimuli, each containing the eleven simulated sound positions was presented (6 blocks, with all 11 stimuli presented once per block in a randomized order, for a total of 66 stimuli presentations). This was done to minimize the possible effect of the duration of GVS. Pauses between blocks were allowed and participants had to maintain the goggles and their position. The possibility of closing their eyes was allowed but participant had to remain alert.

### Analysis

Reported angle values were exported to Matlab (R2019a) and processed using a custom script. While 11 sound source positions were used in the task to keep the same possibilities surrounding the correct answer (one on each side of the correct target), the results from ± 90° sound sources were not analyzed since these positions were our maximum angles, therefore influencing directional bias as subjects were aware that no sound were presented further than ± 90°. We determined the mean error ratio, defined as the total number of mistakes localizing each sound in the concerned quadrant divided by the total number of presentations of the three sound positions in the quadrant. Thus, the mean error ratio for each sound sources located in the left quadrant (− 45°, − 30°, − 20°), center quadrant (− 10°, 0°, 10°), and the right quadrant (45°, 30°, 20°) were calculated for each subject and for each condition (Baseline; Anode right; Anode left). Additionally, we assessed directional bias for each sound source, defined as the proportion trials where the participant chose either a sound source position located to the left or the right to the actual target (ex: a left bias occurs when a participant identifies the sound source as being 20° while the actual target was 30°).

A non-parametric Friedman test was done to compare the three GVS conditions (Baseline; Anode Left; Anode Right) in each quadrant (Left, Center, Right). Friedman test was also conducted to assess the influence of GVS stimulation on directional bias for the 8 sound source positions (− 45, − 30, − 20, − 10, 10, 20, 30, 45). Analysis was performed with IBM Statistical Package for the Social Sciences (SPSS Statistics; Version 29) and the thresholds of significance was delineated at *p* < 0.05. The Wilcoxon rank-sum test with a Bonferroni correction was used as a post-hoc test to reveal differences where applicable.

## Results

**Fig. 2:**
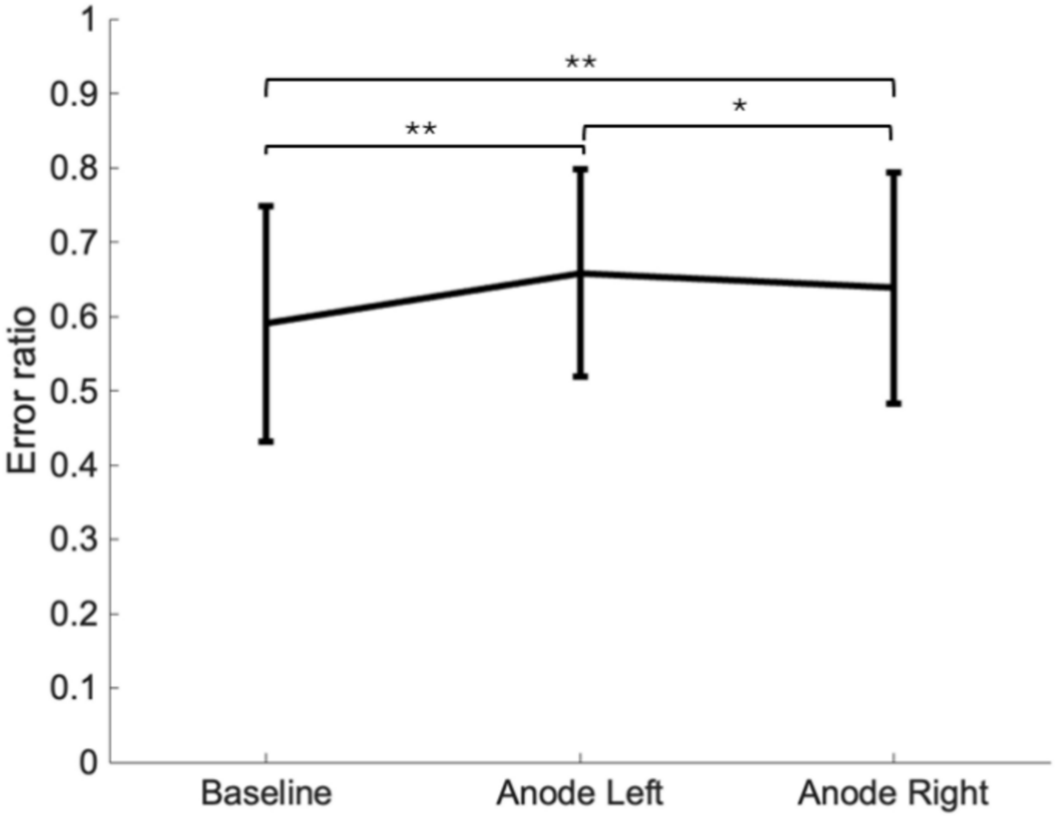
Representation of the average error ratio for each GVS conditions (baseline, Anode Left, Anode Right). A significant difference can be measured between Baseline and Anode Right and between Baseline and Anode Left conditions. A significant difference was also measured between Anode Left and Anode Right conditions. Error bars represent standard deviation. *p < 0.05; **p ≤ 0.001)”.

First, a Friedman test with Bonferroni correction for three GVS conditions (Baseline; Anode Left; Anode Right) across all quadrants was performed to assess the general influence of GVS. Figure 2 reveals a significant impact of GVS [χ^2^(2) = 57,561, *p* < 0.001, w = 0.56 ] which leads to a greater error ratio when GVS is present compared to baseline [Anode left: *p* ≤ 0.001; Anode right: *p* ≤ 0.001]. Moreover, significantly greater error ratios were observed when anode was on the left side compared to anode right condition (*p* = 0.012).

**Fig. 3:**
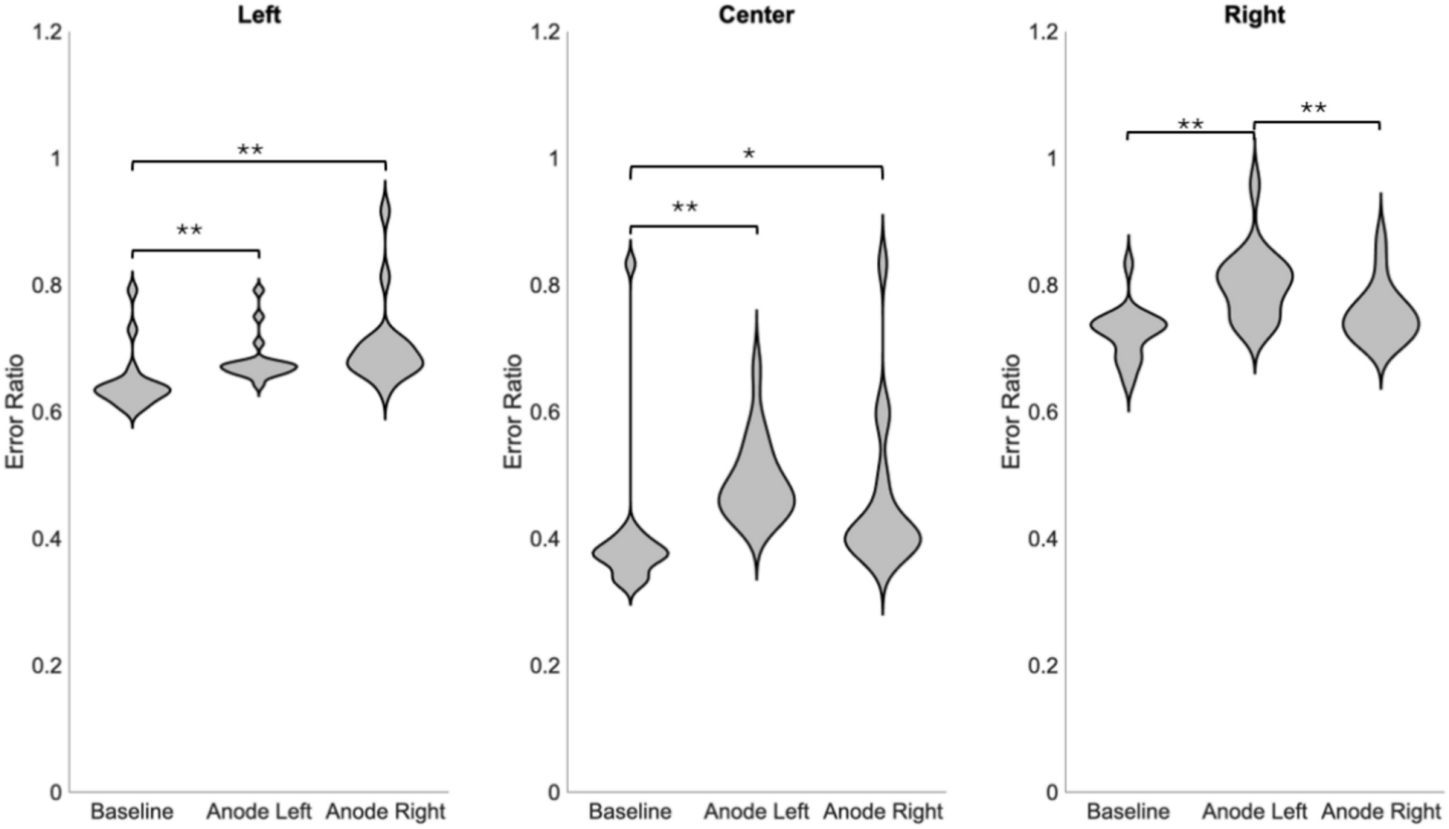
Violin plots representation of the average error ratio for sound sources from the left, the center, and the right quadrant, in the three experimental conditions (baseline, anode right, and anode left) *p < 0.05, **p ≤ 0.001. The Graph shows a significantly greater error ratio for sounds in the left and center quadrants when the anode is placed on both mastoids and for sounds in the right quadrants only when the anode is placed on the left mastoid. Left anodal condition caused greater error ratio compared to right anodal condition only in the right quadrant.

**Fig. 4:**
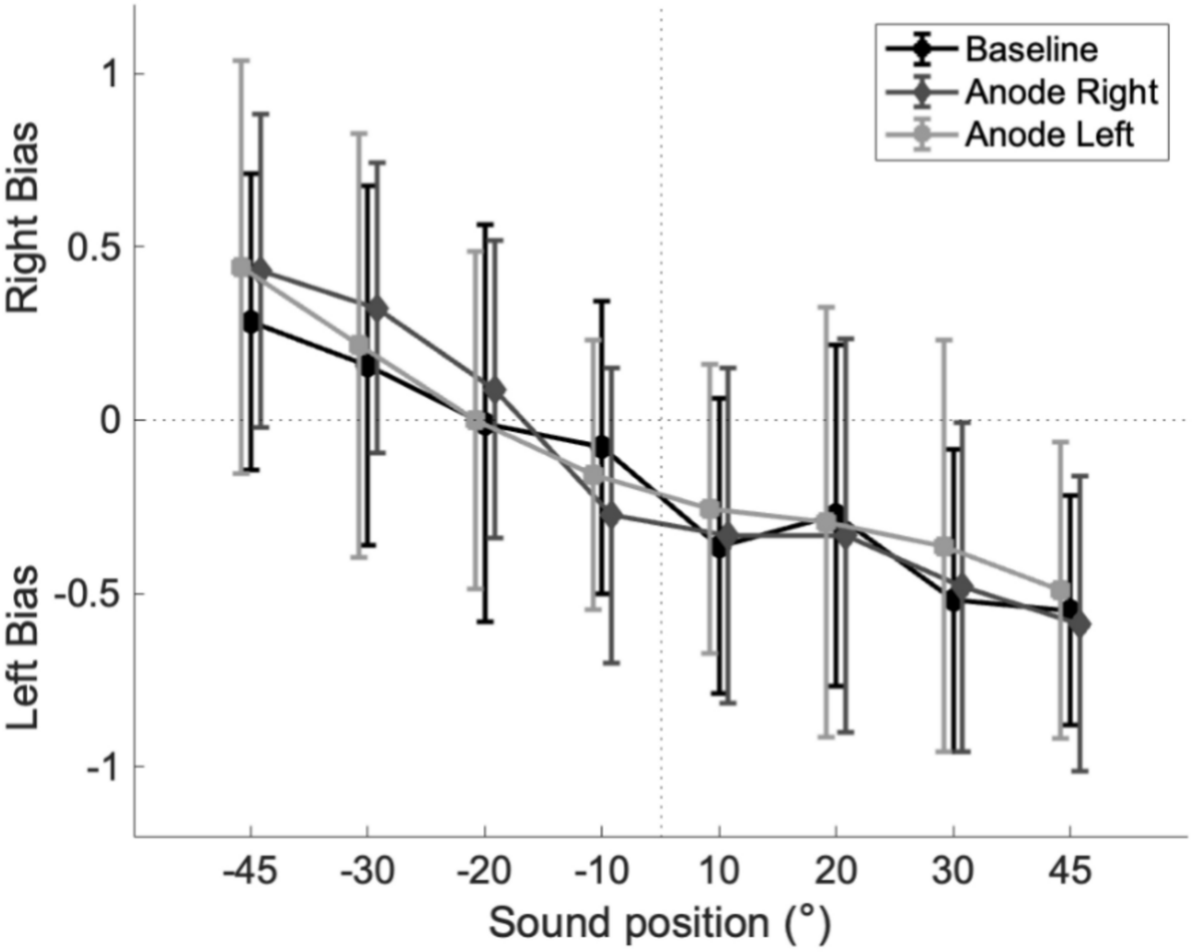
Comparison of the right and left directional bias of the 8 sound source positions between the 3 GVS conditions (baseline, anode right and anode left).

Figure 3 reveals the average error ratio for sounds in the three quadrants. The analysis confirmed a general influence of GVS for sounds in the left quadrant [χ^2^(2) = 24.738, *p* ≤ 0.001, w = 0.73], in the center quadrant [χ^2^(2) = 23,292, *p* ≤ 0.001, w = 0.69] and in the right quadrant [χ^2^(2) = 23.758, *p* ≤ 0.001, w = 0.70]. More precisely, in the left quadrant, Wilcoxon rank-sum test with a Bonferroni correction revealed that right anodal stimulation and left anodal stimulation significantly increased the error ratio compared to the baseline condition (*p* ≤ 0.001); (*p* ≤ 0.001). On the contrary, no significant differences were observed between the right and left anodal stimulations (*p* = 0,910). Moreover, for sounds from the center quadrant, left and right anodal stimulation significantly increased the error ratio compared to the baseline condition (*p* ≤ 0.001); (*p* = 0.039). No significant error ratio for the anode left condition compared to the anode right condition was observed in this quadrant (*p* = 0.077). Finally, for sounds from the right quadrant, left anodal stimulation significantly increased the error ratio, compared to the baseline condition (p ≤ 0.001) and compared to the right anodal condition (*p* ≤ 0.001). No significant difference was observed between the right anodal condition and baseline condition (*p* = 1.00). All comparison were presented in Table 2. Finally, GVS conditions did not significantly influence directional bias of responses [χ^2^(2) = 1.759, *p* = 0.415, w = 0.025 ]. Data regarding directional bias is provided in Fig. 4.

**Table 2:** Raw data comparing mean error ratio during the localization task of sounds in three quadrants (left, center, right) and three GVS conditions (Baseline, Anode left, Anode right) using Friedman test and the Wilcoxon rank-sum test with a Bonferroni correction as post-hoc test **p ≤ 0.001; *p < 0.05.

		Mean error ratio	Std. deviation	*p* value
Left quadrant	Baseline Anode left	0.647 0.681	0.047 0.037	0.001**
	Baseline Anode right	0.647 0.703	0.047 0.067	< 0.001**
	Anode right Anode left	0.703 0.681	0.067 0.037	0.910
Center quadrant	Baseline Anode left	0.399 0.492	0.115 0.064	< 0.001**
	Baseline Anode right	0.399 0.456	0.115 0.120	0.039*
	Anode right Anode left	0.456 0.492	0.120 0.064	0.077
Right quadrant	Baseline Anode left	0.725 0.801	0.042 0.056	< 0.001**
	Baseline Anode right	0.725 0.755	0.042 0.045	1.00
	Anode right Anode left	0.755 0.801	0.045 0.056	< 0.001**

## Discussion

The present study demonstrates the modulatory effect of an induced vestibular asymmetry using direct current GVS on the ability to localize sounds in space. More specifically, the results revealed that the position of the anode asymmetrically affects the ability to localize sounds accurately. Indeed, right anodal and left cathodal stimulation caused a significantly greater error ratio regarding sounds in the center quadrant and the left quadrant whereas left anodal and right cathodal stimulation caused a significantly greater error ratio for sounds in all quadrants.

These results support previous findings suggesting an influence of vestibular afferent inputs on sound localization^10,11^, while also adding specificity by demonstrating an asymmetrical influence based on the position of the anode.

Several studies have demonstrated the activation of a network composed of areas predominantly located in the bilateral temporal-parietal cortex following GVS and caloric vestibular stimulation, two methods known to induce peripheral vestibular asymmetry^16–19^. For example, Karim et al.^19^ demonstrated that cold caloric stimulation induced a contralateral activation of the temporoparietal cortex that is greater when the left ear is stimulated. Interestingly, among several activated areas, Bucher et al.^18^ observed bilateral cortical activation during GVS in Brodmann area 22 (BA 22), located in the superior temporal gyrus, and Heschl’s gyrus. These areas are well known to be involved in auditory processing^20,21^. Brunetti et al.^20^ used a passive localization task with different sounds positions (-90°, -45°, 0°, + 45°, + 90°) virtually generated in an anechoic room and they analyzed MRI and MEG data of neurotypical subjects. Results revealed significant activation in a complex neural circuit that included Heschl’s gyrus and the superior temporal gyrus (STG). A more recent study that investigated brain activation with functional near-infrared spectroscopy (fNIRS) during a sound localization task brings more precision in the area activated based on the sound source position^21^. They found that sounds at + 90° and − 90° generated an increased oxy-Hb response in BA22 and BA42, bilaterally, as well as in part of Wernicke’s area in the hemisphere contralateral to the stimuli, while no oxy-Hb responses were observed for sounds at 0°. Therefore, GVS impacts on sound localization may be on a central processing level where it modulates the activation of these cortical areas. However, this interpretation needs to be considered with precaution since to our knowledge, no other research has evaluated cortical activation during sounds at the positions tested in the present study. Future studies using brain imagery techniques will need to be conducted regarding sounds in other positions along the azimuth plane.

A remaining question is why significant error ratios for sounds in all quadrants were observed in the left anodal stimulation condition. As mentioned earlier, activation of the contralateral hemisphere following cold CVS is greater when the left ear is stimulated. It is therefore possible that the induced asymmetric cortical activation could be greater in the left anodal stimulation condition and that, as a result, difficulties not only occurred at the right quadrant but also expanded to the center quadrant and left quadrant. More specifically, as the vestibular dominant hemisphere depends on the handedness and that the vast majority of the present study sample is right-handed, a left anodal stimulation could potentially have greater influence on the right hemisphere^22^. Additionally, studies on brain lesions have found that participants with lesions in the right hemisphere presented sound localization deficits that were more pronounced and generalized in the auditory field compared to lesions in the left hemisphere^23^. Therefore, impaired localization of sounds in the right quadrant as well as sounds in the center quadrant following left GVS could be explained by the greater impact of GVS on the right hemisphere’s ability to localize sound sources and its importance in right-handers. However, a study evaluating brain regions activated following GVS found symmetrical activation in both hemispheres regardless of the anode position^16^. While the results from this study need to be taken with caution since data from only 6 subjects divided into three groups were analyzed, further investigation into GVS cortical activation patterns is warranted.

To our knowledge, only one previous study has investigated audio-vestibular interaction using GVS. Zanchi et al.^4^ asked participants to walk toward loudspeakers, the position of which was encoded briefly prior to walking with or without GVS. Contrary to the results obtained in the current study, they observed that the presence of GVS did not modify performance in localizing the sound source. One possible explanation lies in the type of current used during GVS. Indeed, they were using stochastic vestibular stimulation, a random or pseudo-random electrical noise that induces no consistent or directional illusory self-motion contrary to the direct current used in the present study that is formed of a constant current signal. Another possible explanation lies in the task used in the Zanchi et al.^4^ where the walking task used may have involved other sensory and motor afferents limiting the influence of vestibular perturbation. Our experimental paradigm seems to isolate better vestibular contributions since we used a stationary task.

This contrasts with the stimulus used in the present study and previous literature that used caloric and rotational stimulation^10,11^. Indeed, caloric stimulation and direct current GVS are vestibular stimulus known to consistently induce a sense of self-motion^13,24^. In the present study, a directional illusion of self-motion was perceived by our participants, as opposed to a generic weak dizziness sensation as described in Zanchi et al.^4^ Therefore, this supports our initial hypothesis that a directional illusory motion perception is required to update spatial body representation. A previous study also supports this hypothesis, with different methodology. Paromov et al.,^8^ studied healthy participants while performing the Fukuda stepping task (walking in place eyes closed) with a sound source at different azimuthal positions. During this task, participants were not aware of body rotations and therefore did not update body representation in space, which led to ignoring accurate auditory cues when localizing sound sources in space. In the present study, the participants updated the body in space representation based on the illusory self-motion perception induced by direct current GVS. However, the auditory cues of the sound sources remained unchanged (under headphones), which led to a mismatch in the spatial representation relative to the body midline.

Another possible explanation for the discrepancy between Zanchi et al.^4^ and the present study is the presence of GVS during spatial encoding. In the present study, the sound stimulus and GVS signal were presented continuously and simultaneously. Conversely, participants in Zanchi et al.^4^ were exposed to a ten-second GVS stimulation, but the auditory was simultaneously presented with the stimulation for only 0.5 s. Therefore, the short overlap of the vestibular stimulation and acoustic stimulation used may not be sufficient to influence auditory processing^4^. Therefore, audio-vestibular interaction with constant vestibular perturbation during dynamic tasks remained unexplored and could be an interesting avenue for future studies.

The influence of hemispheric biasing of attentional mechanisms on the results obtained in the present study remains an open question. It has been demonstrated that brain stimulation using transcranial direct current stimulation (tDCS) modulates attentional control processes^25^. More precisely, Duecker et al. demonstrated that an interhemispheric imbalance of the posterior parietal cortex (PPC) using tDCS modulates attentional processes in orienting tasks. Since GVS is known to activate the PPC among other brain areas^20^, the brain activation pattern influencing attentional mechanisms is a plausible factor that could explain the results obtained here. However, the posterior parietal cortex is also known to be implicated in central vestibular processing such as spatial representation and the encoding of precise self-motion perception^26^.

Therefore, an attentional bias cannot, in and of itself, explain the anodal effect observed in the present study. In the future, it would be interesting to explore the impact of interhemispheric imbalance mediating attentional process using brain stimulation methods, such as the tDCS, in normal participants and vestibular impaired participants, on the auditory task used here.

In the literature a bias toward central position (0°) was observed during auditory localization task when visual cues were provided^27^. In the present study participants had to fixate a light positioned centrally. Therefore, it is not surprising that a bias toward more central position was observed in our study. However, it seems that GVS did not influence significantly the directional bias, but did increase the error ratio.

Five principles limitations in our approach need to be addressed. First, possible discomfort following a long period of galvanic vestibular stimulation forced us to create a limited number of sound positions and therefore led to considerable gaps between sound sources within quadrants. In addition, even though several measures were taken to ensure normal vestibular function such as recruiting a relatively young cohort, questions on the history of vestibular symptoms and, screening of horizontal vestibular canals function using vHIT, it remains impossible to rule out otolithic or vertical canal impairment since those structures were not objectively evaluated. Also, even though, many participants of the study reported a self-motion perception during GVS and that self-motion perception induced by direct current GVS is well known in the literature, the presence of motion perception was not systemically asked by the experimenter. Future studies should consider measuring motion perception to ensure that all participants were in fact perceiving motion. Additionally, no Sham stimulation was used to control for a placebo effect. Even though the non-placebo effect of GVS is reinforced by the different influences based on the anodal position, sham stimulation remains important and should be considered in future GVS studies. Finally, participants were asked to report the position of the sound source verbally. Even though participants were trained to identify sound positions before the experimental tasks, a subjective bias remains possible. Future studies could compare different objective methods to measure the performance of localized sound sources under earphones.

## Conclusion

The present study demonstrates that spatial auditory processing can be altered by galvanic vestibular stimulation in normal healthy individuals. This modulation may be a matter of central processing, where a disparity in the hemispheric brain activation of auditory areas could be occurring following galvanic vestibular stimulation. We also demonstrate for the first time that sound localization performance varies according to the position of the anode during galvanic vestibular stimulation. Future studies should investigate how this auditory-vestibular interaction transposes dynamic and naturalistic tasks, such as walking.

## Data Availability

The data that support the findings of this study are available from the corresponding author, upon reasonable request.
